# Quality of Life in Chronic Ketogenic Diet Treatment: The GLUT1DS Population Perspective

**DOI:** 10.3390/nu11071650

**Published:** 2019-07-19

**Authors:** Costanza Varesio, Ludovica Pasca, Stefano Parravicini, Martina Paola Zanaboni, Elena Ballante, Silvia Masnada, Cinzia Ferraris, Simona Bertoli, Anna Tagliabue, Pierangelo Veggiotti, Valentina De Giorgis

**Affiliations:** 1 Department of Child Neurology and Psychiatry, IRCCS Mondino Foundation, Via Mondino 2, 27100 Pavia, Italy; 2 Department of Brain and Behavioral Sciences, University of Pavia, 27100 Pavia, Italy; 3 BioData Science Center, IRCCS Mondino Foundation, 27100 Pavia, Italy; 4 Computational Mathematics and Decision Sciences, University of Pavia, 27100 Pavia, Italy; 5 Human Nutrition and Eating Disorder Research Center, Department of Public Health, Experimental and Forensic Medicine University of Pavia, 27100 Pavia, Italy; 6 International Center for the Assessment of Nutritional Status (ICANS), Department of Food Environmental and Nutritional Sciences (DeFENS), University of Milan, Via Sandro Botticelli 21, 20133 Milan, Italy; 7 Pediatric Neurology Unit, Vittore Buzzi Hospital, Via Lodovico Castelvetro 32, 20154 Milan, Italy; 8 Biomedical and Clinical Sciences Department, Luigi Sacco Hospital, University of Milan, via G. B. Grassi 74, 20157 Milan, Italy

**Keywords:** ketogenic diet treatment, GLUT1 deficiency syndrome, health related quality of life, PedsQol 4.0

## Abstract

Background: Glucose transporter type 1 deficiency syndrome (GLUT1DS) is a rare, genetically determined neurological disorder, for which Ketogenic Diet (KD) represents the gold standard life-long treatment. The aim of this study is to investigate health related quality of life in a well characterized cohort of patients affected by GLUT1DS treated with KD, evaluating factors that can influence patients’ and parents’ quality of life perception. Methods: This is a double center exploratory research study. A postal survey with auto-administrable questionnaires was conducted among 17 subjects (aged 3–22 years) with diagnosis of GLUT1DS, receiving a stable KD treatment for more than 1 year. The Pediatric Quality of Life Inventory (PedsQL) 4.0 Generic Core Scales was adopted. Clinical variables analyzed in relation to quality of life were frequency of epileptic seizures and movement disorder since KD introduction, presence of intellectual disability (ID), and KD ratio. Results: Quality of life global scores were impaired both in parents’ and children’s perspectives, with a significant concordance. Taking into consideration subscales, the average was 64.17 (range 10–100) for physical functioning, 74.23 (range 30–100) for emotional functioning, 62.64 (range 10–100) for social functioning, and 56 (range 15–92) for school functioning. Conclusions: In patients with GLUT1DS the quality of life perception is comparable to that of other patients with chronic disease. In our sample, the presence of movement disorder seems to be a crucial element in quality of life perception.

## 1. Introduction

Glucose transporter type 1 deficiency syndrome (GLUT1DS) is a rare, genetically determined, treatable neurological disorder. It is caused by a mutation in the *SCL2A1* (solute carrier family 2, facilitated glucose transporter member 1) gene, on chromosome 1p35-31.3,1 which is, so far, the only gene known to be associated with this condition. The consequence of haplo-insufficiency is decreased availability of glucose to the brain and resultant encephalopathy [1,2]. 

Currently, GLUT1DS is recognized to have a wide spectrum of manifestations with variable degrees of severity and different timing of presentation through a patient’s life. Common manifestations are represented by microcephaly, cognitive impairment, epilepsy, and paroxysmal exercise-induced dyskinesia [3,4,5,6,7,8].

The Ketogenic Diet (KD) has been used since 1921 for the treatment of refractory epilepsy. From the first reports of GLUT1DS in 1991, the classic KD has been consistently described as the gold standard therapy according to disease characteristics and symptoms: ketone bodies can cross the blood brain barrier and can be used as alternative fuel for brain metabolism [9,10,11]. For patients with GLUT1DS, KD efficacy is proven both in reduction of epileptic seizures and movement disorder [12,13,14], as well as in intellectual disability recovery [15,16,17].

The updated international consensus for its use, states that ketogenic diet therapy should be strongly considered in a child with whom two anti-epileptic drugs (AED) have failed and that KD is the treatment of choice for two specific disorders of brain metabolism, GLUT1DS and pyruvate dehydrogenase deficiency (PDHD). According to the international consensus study group, consideration should be given to discontinuing KD after 2 years for cases in which there has been benefit but long-life diet duration is likely necessary for GLUT1DS [18]. 

In general terms, the KD diet is a high-fat, low carbohydrate, adequate-protein diet with a high ketogenic ratio (fat: carbohydrates + protein in weight) [19]. Despite its proven effectiveness, adherence to KD is extremely demanding both for patients and for their families. More liberal versions of dietary therapies exist beyond the classical KD. Modified Atkins diet (MAD) has been described as a less strict alternative in patients with GLUT1DS with positive effects both on seizures and on MD [15,20]. Haberlandt et al. [21] suggested that MAD might be an effective dietary treatment for GLUT1DS in patients with missense mutations. More precisely, patients with missense mutations with preserved residual function of GLUT1 transporter between 50% and 75% usually show a mild phenotype [22], thus supporting the hypothesis is that in that specific group of patients a “milder” ketosis may be sufficient for symptoms control [21]. 

It has been estimated that in children, brain energy demand is three to four times higher than in adults [23], and the ability of a child brain to extract and utilize ketone bodies is four times higher than in adults [24]. Patients with GLUT1DS should start KD as early as possible and should remain on diet at least until adolescence. If no serious side effects develop, recommendation is to continue classic KD in children and symptomatic adults, whereas a less strict regimen such as MAD can be proposed to adolescents and adults with minimal symptoms [11]. 

In recent years, there is an increasing awareness and interest in social, psychological, and behavioral well-being in patients with chronic conditions. Definition of quality of life (QoL) is not univocal. When considering its definition, we usually refer to the World Health Organization definition, that is “the individual’s perceptions of their position in life in the context of the culture and value systems in which they live, and in relation to their goals, expectations, and concerns” [25]. 

Many standardized tools and measures have been developed and well validated to try to capture the impact of disease on QoL—the so-called Health Related Quality of Life (HRQOL). These include generic measures such as the PedsQL (Pediatric Quality of Life Inventory). More in detail, the Pediatric Quality of Life InventoryTM (PedsQLTM) 4.0 Generic Core Scales is a generic HRQOL instrument for children, adolescents, young adults and their parents, that is applicable both for healthy populations as well as for populations with acute and chronic conditions [26,27]. 

QoL is an important outcome measure for children with chronic health conditions and their families. In the last few years, there has been growing interest in quality of life in patients with refractory epilepsy treated with KD, to improve compliance to the treatment since poor adherence to the treatment could worsen clinical conditions. For instance, ad hoc instruments to measure the impact of KD on caregivers’ and patients’ lives have been developed [28,29]. However, none of them refer to GLUT1DS patients.

The aim of this study is to assess HRQOL in a well-characterized cohort of patients affected by GLUT1DS in treatment with KD. We also investigated potential influencing factors on children’s and parents’ QoL perception. These possible influencing factors are both related to the pathology itself (presence of epilepsy or movement disorder, presence of intellectual disability), as well as to the treatment with KD. 

## 2. Materials and Methods 

### 2.1. Study Design and Setting

This is a double center exploratory study, conducted by a postal survey among patients (children, adolescents and young adults) affected by GLUT1DS and their families.

### 2.2. Subjects

After approval from our Ethical Committee (P-20190033749), we enrolled 17 subjects with definite diagnosis of GLUT1DS followed as inpatients or outpatients at the Child Neurology Unit in IRCCS Mondino in Pavia and at the Pediatric Neurology Unit in Buzzi Children’s Hospital in Milan, receiving a stable KD treatment for more than one year.

As GLUT1DS is a rare disease, we used these broad inclusion criteria, and there was no sample size calculation.

Exclusion criterion were represented by lack of stability of clinical conditions during the last month before the administration.

We sent an information letter on study background and questionnaire administration guidelines, adopted from the PedsQL™ website—Italian validated form (Italian version—http://pedsql.org)—to all study participants. Questionnaires were auto administered by patients and parents. Parents were especially advised to let their child fill in the questionnaire separately, without comparing their results. 

### 2.3. Instruments

The Pediatric Quality of Life Inventory (PedsQL) 4.0 Generic Core Scales is designed for patients aged 2–25. According to patients’ ages, different versions were administered. In particular, the PedsQL™ Scales are comprised of parallel child self-report and parent proxy-report formats. Self-report form includes ages 5–7 (young child), 8–12 (child), 13–18 (adolescent), and 18–24 (young adults). The parent proxy-report includes ages 2–4 (toddler), 5–7 (young child), 8–12 (child), 13–18 (adolescent), and 18–24 (young adults).

The PedsQL 4.0 Generic Core Scales, Italian validated form, is a questionnaire consisting of 23 items, categorized into four subscales: physical, emotional, social, and school functioning. The responses ranged from 0 (never a problem) to 4 (almost always a problem). All items were linearly converted and transformed into 0–100 scales. Higher scores represented better HRQOL. It is worth noting that there are no definitive ‘cutoff’ points that delineate ‘good’ QoL from ‘poor’ QoL. However, various studies have tried to differentiate good from impaired QoL. Cut offs for this study were chosen to be based on the study of Kim et al. [30], which put a cut off of 70 to separate patients with high QoL from ones with impaired QoL and on the study by Varni and Limbers [26], who identified child self-reported total score of 69.1 and parent proxy reported total scores of 65.4 as “meaningful cut off points for impaired HRQol,” at one standard deviation below the average PedsQL score for healthy children. 

The reliability and validity of PedsQL generic core scales have been well established in healthy and sick populations [26,27,31,32,33]. 

### 2.4. Statistical Analysis

The statistical analysis performed on the data were based on two stages: a preliminary descriptive analysis and a correlation analysis.

In the descriptive part average and standard deviation values were evaluated for each considered variable to describe the collected data. 

The Spearman’s coefficient was used to measure the correlation among variables and the corresponding *p*-values for the significance of the correlation are reported. The correlation analysis was performed taking into account the global score and then the subscores contained in the surveys.

A further correlation analysis was carried to evaluate whether an agreement was present among the child and the parent scores.

The statistical analysis was performed using R 3.5.1 (R Core Team (2013). R: A language and environment for statistical computing. R Foundation for Statistical Computing, Vienna, Austria).

## 3. Results

### 3.1. Study Population

We recruited 17 patients, nine males and eight females (sex ratio male/female 1:1), aged 3–22 years old (average 10 years old), with established diagnosis of GLUT1DS.

For each patient included in the study information such as frequency of epileptic seizures and movement disorder (MD) since KD introduction, presence of intellectual disability (ID), and KD ratio were collected and analyzed in relation to QoL (Table 1 shows the main characteristics of the study population).

As far as seizure frequency is concerned, patients in our sample have been seizure free since the introduction of KD, with the exception of one patient who has epileptic seizures less than monthly. Only two patients are still on AED.

Taking into consideration MD, 53% of patients (nine patients out of 17) still have MD, with variable frequency (less than monthly in four patients, monthly in one patient, weekly in one patient, daily in three patients). 

As far as ID is concerned, 35% of our patients (six out of 17) have no ID or borderline IQ, 47% (eight out of 17) have mild to moderate ID and 11.7% (two out of 17) show severe ID. Evaluation of intelligence quotient (IQ) was not available only for one patient.

All patients in our study are treated with classical KD (4:1, 3:1, or 2:1 fat to non-fat ratio), with adequate compliance and no serious adverse events reported. In detail, 12% (two out of 17) are treated with a 4:1 regimen, 65% (11 out of 17) with 3:1, and 23% (four out of 17) with 2:1, according to a standardized protocol [34]. 

### 3.2. Health Related Quality of Life

Regarding the families, 17 parents (mothers or fathers) (100%) completed the parent proxy report. The mean PedsQL score for total functioning was 64.59 (range 43–94, SD 15.24). Taking into consideration subscales, the average was 64.17 (range 10–100, SD 21.96) for physical functioning, 74.23 (range 30–100, SD 18.86) for emotional functioning, 62.64 (range 10–100, SD 22.23) for social functioning, and 56 (range 15–92, SD 19.78) for school functioning.

**Figure 1:**
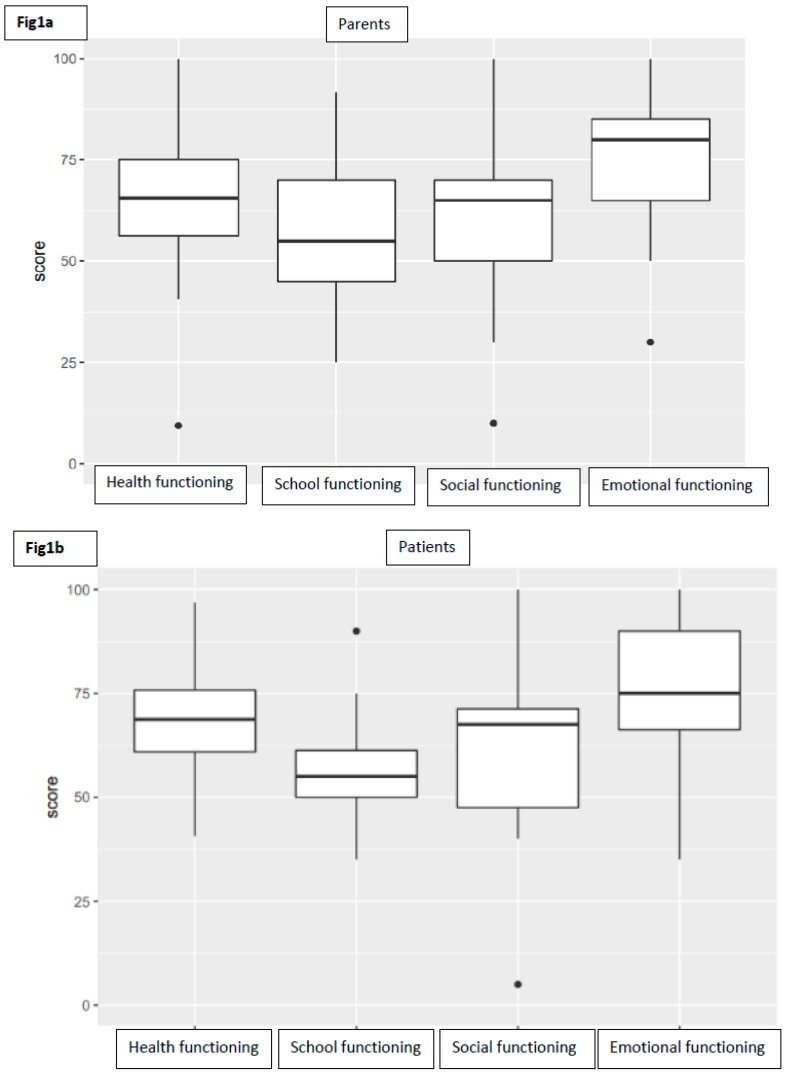
Boxplots representing the distribution of subscores of parents (**a**) and patients (**b**).

As far as child self-report is concerned, 12 patients completed their own questionnaire (four patients were too young, another one was unable to understand questions). The average PedsQL score for total functioning was 65.08 (range 50–97, SD 12.74). Taking into consideration subscale scores, the average was 67.58 (range 41–97, SD 14.87) for physical functioning, 73.75 (range 35–100, SD 18.72) for emotional functioning, 61.25 (range 5–100, SD 22.64) for social functioning, and 57.08 (range 35–90, SD 14.20) for school functioning (Figure 1).

### 3.3. Factors Influencing HRQOL

QoL perception by patients and parents was analyzed accordingly to identify possible influencing factors, related both to treatment with KD and to the underlying pathology itself. Table 2 summarizes *p*-value results of correlation analysis.

Taking into consideration sample demographics, we identified a significant correlation between patients’ age and presence of worries about the future (subdomain 5 of emotive functioning) in parents’ report (*p*-value 0.05, correlation coefficient 0.47), as well as troubles in getting along with others (subdomain 1 of social functioning) in parents’ report (*p*-value 0.02, correlation coefficient 0.54).

As far as KD is concerned, we observed a significant correlation between KD ratio and frequency of MD (*p* value 0.045, correlation coefficient −0.49), presence of somatic complains (subdomain 7 of health functioning) in parents’ report (*p*-value 0.009, correlation coefficient 0.6), trouble in paying attention at school or work (subdomain 1 of school functioning) both in parents’ and children’s reports (*p*-value for parents’ report 0.03, correlation coefficient for parents’ report 0.52; *p*-value for children’s report 0.006, correlation coefficient for children’s report 0.73), trouble in keeping up with studies or work (subdomain 3 in school functioning) in parents’ report (*p* value 0.033, correlation coefficient 0.51).

Furthermore, we searched for a possible correlation between HRQOL and typical characteristics of GLUT1DS.

Considering MD, we found it to be significantly related to global health functioning in parents’ report (*p*-value 0.046, correlation coefficient −0.48), global school functioning in parents’ report (*p*-value 0.002, correlation coefficient −0.68), trouble in sleeping (subdomain 4 of emotional functioning) both in parents’ and in children’s reports (*p*-value in parents’ report 0.006, correlation coefficient in parents’ report −0.63; *p*-value in children’s report 0.016, correlation coefficient in children’s report −0.67), ability in paying attention at school/work (subdomain 1 of school functioning) both in parent’s and in children’s reports (*p*-value in parents’ report 0.031, correlation coefficient in parents’ report −0.52; *p*-value in children’s report 0.026, correlation coefficient in children’s report −0.643), keeping up studies or work (subdomain 3 of school functioning) in parents’ report (*p*-value 0.01, correlation coefficient −0.60); missing school or work (subdomain 5 of school functioning) in parents’ report (*p*-value < 0.01, correlation coefficient −0.69).

Intellectual disability was found to be correlated with doing chores around the house (subdomain 6 of physical functioning) both in parents’ and in children’s reports (*p*-value in parents’ report < 0.009, correlation coefficient 0.62; *p*-value in children’s report 0.01, correlation coefficient in children’s report 0.69), trouble in making friends (subdomain 2 of social functioning) in parents’ report (*p*-value 0.02, correlation coefficient 0.55), being teased by others (subdomain 3 of social functioning) both in parents’ and in children’s reports (*p*-value for parents’ report 0.01, correlation coefficient in parents’ report 0.57; *p*-value for children’s report 0.007, correlation coefficient 0.72), memory problems (subdomain 2 of school functioning) in parents’ report (*p*-value 0.05, correlation coefficient 0.49), and trouble in keeping up with studies or work (subdomain 3 of school functioning) in children’s report (*p*-value 0.006, correlation coefficient 0.73);

### 3.4. Concordance between Self and Proxy Reports

**Figure 2:**
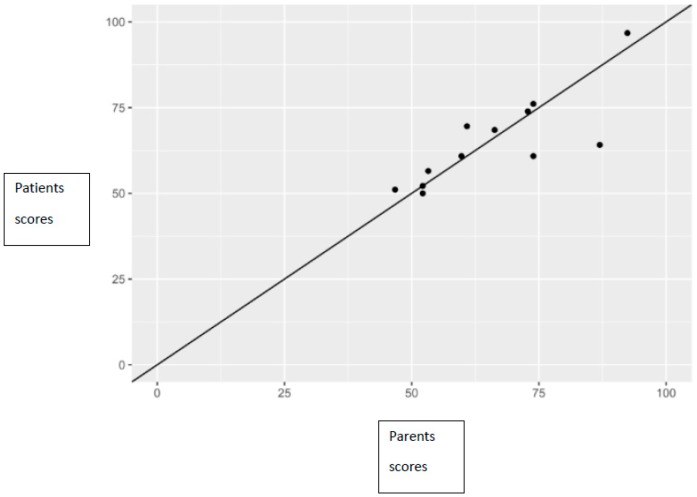
Relationship between parents’ and patients’ scores, along with the line of perfect concordance.

There was a statistically significant positive correlation between total PedsQL scores reported by children and those reported by their parents, which was observed both in total scores (correlation coefficient 0.81), as well as in the subscale score (correlation coefficient for health functioning 0.65, correlation coefficient for emotional functioning 0.73, correlation coefficient for social functioning 0.97, correlation coefficient for school functioning 0.61) (Figure 2).

## 4. Discussion

To the best of our knowledge, this is the first study that systematically evaluates the impact of KD in patients with GLUT1DS. Previous studies [35,36] have tried to define the impact of KD on patients and families through questionnaires filled in by patients, caregivers, and clinicians. In particular, parents’ satisfaction with diet was evaluated through qualitative assessment (very satisfied, satisfied, unsatisfied, or very unsatisfied); no quantitative assessment through standardized questionnaires was outlined. In these studies, families and patients showed, overall, high levels of satisfaction for neurological symptoms, whereas they showed concern about long-term diet continuation.

In this study, we investigated HRQOL in a cohort of pediatric to young adults with GLUT1DS treated with classical KD, considering factors that can possibly influence QoL perception.

Patients with GLUT1DS represent a particular group of patients following KD: in addition to a chronic neurological condition, they present further difficulties associated with a restrictive dietary regimen with classic KD that should be maintained life-long.

Although the determination of side effects was not considered as part of this study, we can say that in this specific subset of patients no significant side effects deriving from KD were observed, except for modest transient gastro-intestinal symptoms. Studies aimed at assessing tolerability of KD show that patients treated with KD may have short term side effects (mostly represented by mild gastro-intestinal symptoms) and long term side effects (represented by kidney stones, decreased linear growth, excessive weight loss, vitamin deficit, and alterations in body composition). However, most of them are not serious and are easily preventable and treatable with a close follow up [34,37,38,39].

Lack of palatability of ketogenic food and qualitative and quantitative restrictions in meals preparation can be burdensome. Each ingredient needs to be weighed per gram, resulting in a long time spent for meal preparation; the overall treatment should be continuously monitored and tailored to the individual patient. A great aid to families is given by applications available for smartphones, where appropriate count of nutrients can be easily calculated for each meal and recipes approved by dieticians can be shared. Adherence to KD implies important limitations in participation to daily social activities, not only for patients but also for their families. Despite these difficulties, our experience suggests that in the specific set of GLUT1DS patients, compliance has proven to be better than in other conditions for which KD is indicated [13], likely because of the efficacy of the treatment on symptoms. However, in a recent study by Bekker et al. [40] a small cohort of patients failing on KD because of inefficacy (poor effect despite adequate ketosis), as well as intolerance and an inability to attain ketosis has been described.

Since KD is an individualized treatment, higher ratios are prescribed in patients with more severe phenotype, in whom symptoms cannot be controlled with a lower ketogenic ratio [11]. According to this statement, from our data analysis, we observed a significant relation between severity of MD and higher KD ratio, possibly meaning that in these patients symptoms control is more complex.

According to parents’ report, patients with higher KD ratio have more somatic complaints, this is in line with the idea that KD with higher ratio is more difficult to manage, less palatable, and more difficult to tolerate.

Overall, we found that QoL scores were impaired both in parents’ and children’s perspectives. Parents proxy report global results were in line with the cut off defined by Varni and Limbers [27] as “meaningful cut off points for impaired HRQoL,” thus revealing a “borderline” QoL perception. This result is consistent with parents’ perception of living with children with chronic medical conditions as reported by Kim et al. [30], who showed that patients’ QoL resulted in being affected in a superimposable way to what was expected for children with chronic illnesses. Average results in various subdomains are affected almost homogeneously. Since no other data on QoL of GLUT1DS patients are available to be compared with our results, it may be interesting to mention studies on QoL of patients with an inborn error of metabolism (IEM). Importantly, Eminoglu et al. [41] studied QOL in children with IEMs whose treatment involves a restrictive diet, underlining how as negative effects of the disease increase, the QoL of IMD patients and their parents decreased in terms of emotional, physical, and cognitive function. In patients with phenylketonuria (PKU) a negative impact of PKU on a patient’s life, in particular the emotional impact, was evidenced across all age groups in the study of Bosch et al. [42]. Specifically, patients with milder PKU reported a lower social and practical impact of dietary restriction. The study of Eggink et al. [43] showed that HRQOL in children with IEM and co-existent movement disorder is significantly reduced compared to other chronic disorders. Delay in adaptive functioning and a more severe movement disorder were associated with a lower HRQOL.

In our sample we found a significant relationship between the presence of MD and health and school functioning subscales. This means that the worse the MD, the worst the parents’ reported performance in health and school functioning. In particular, the subdomains primarily significantly affected are represented by the ability of paying close attention and following school activities. In addition, frequent school absences have been identified as significantly related to the presence of MD. Moreover, we identified both for parents and for children that the presence of MD relates significantly with the presence of sleep disorders. We can hypothesize that not only symptoms of GLUT1DS, but also KD itself may affect quality of sleep. To the best of our knowledge, the effects of KD on sleep have not been clearly elucidated yet. In their study on the effects of KD in patients with intractable epilepsy, Hallbook et al. [44] concluded that KD decreases sleep and improves sleep quality, in particular by increasing REM sleep in children with therapy-resistant epilepsy. They also observed improvement in seizure frequency, seizure severity, attentional behavior, and QoL and a significant correlation between increased REM sleep and QoL at 3 months. However, it is extremely difficult to refer these data to the population of patients with GLUT1DS, and in particular to our population, where sleep disorder seems to be related to MD.

Focusing on ID, as intuitively expected, patients with ID have more problems in the field of social functioning (in particular represented by difficulties in relating with peers). As far as school functioning is considered, a concern for frequent school absences emerged from parents’ reports, whereas significant difficulties in doing their homework both in school and at home emerged in children’s reports.

In our sample nothing can be said about the role of epileptic seizures as they are sporadically present in only one subject, the same is true for AED treatment, which is still ongoing only for two patients.

In parents’ reports a significant relationship between child age, presence of concern regarding health conditions, and difficulties in interaction with peers was found. This evidence can be easily interpreted: from late childhood to adolescence, awareness of their condition as chronic patients emerges. On the other hand, difficulties in coping with meal preparation and restrictions lead to limitations in sharing activities with peers.

Although not statistically significant, we consider it important to emphasize that the score obtained in emotional functioning seems to be mildly impaired both for parents and patients. This is consistent with previous observation by Pearson [5], who noted that adaptive socialization skills (in particular the fact of being well related, socially outgoing, and friendly) are strengths for the group of patients with GLUT1DS. Moreover, in a long term follow-up in 12 patients, Alter [45] found out that nine of them exhibited exceptional empathy for others. We believe that this peculiar aspect deserves to be further investigated in future studies.

A noteworthy datum is the significance of agreement between parents’ and children’s answers. We observed a concordance both in the global score as well as in various subdomains. In particular, the interclass correlation coefficient was higher in social and emotional functioning. This differs from what was outlined by Eiser and Varni [46], who claimed that it is generally accepted (but not universally observed) that parents of children with chronic conditions tends to rate their children’s HRQL less highly than what children do themselves, and that concordance is greater in areas of observable functioning than in areas of not observable functioning. One reason for this difference could be that children with greater cognitive impairment, that could be the same who did not perceive the items proposed as problematic, were not able to complete the questionnaire, while their parents did. On the other hand, this excellent concordance may rely on particular sensitivity and attention paid by parents in looking over outside home activities or relationships involving their children, due to the needs that go along with KD. Moreover, the fact that parents, together with children, often give up social activities because of the KD, confers them extreme awareness of children’s feelings and of innermost illness effects on QoL. Overall, this moderate-to-strong agreement between children and parent ratings supports the usefulness of parents’ proxy reports. As stated by Upton et al. [47], when measuring HRQL in children, it is desirable to get reports both from children and parents; however, according to our data, when child responses are unavailable or untrustworthy, parental responses can be considered a reliable indicator of the health status of patients with GLUT1DS in life-long treatment with KD.

These results have also implications for the management of follow-up. As stated by Costello et al. more than 30 years ago [48], psychosocial health has become the “new hidden morbidity” in pediatric health care. As already highlighted by Seid et al. [49], if healthcare providers knew in advance which children are at greater risk for future health related problems, then healthcare resources could be proactively targeted specifically to those children.

We believe it is essential to dedicate special attention and hopefully improve our knowledge of the psychological profile of patients with GLUT1DS in order to appropriately meet developmental, educational, and functional needs together with medical needs. We believe that granting specific and constant psychological support, even by creating group counseling for patients and their parents, embracing the cooperation with families’ associations could be useful to share difficulties and burdens posed by disease and KD, improving QoL.

Finally, our results must be read and interpreted considering certain limitations. First of all, the population is small. We compared parents and child ratings on a single measure of PedsQL. To better define the impact of KD on quality of life perception we propose conducting a prospective study that includes an evaluation of HRQL at the time of diagnosis, i.e., before the introduction of KD.

Secondly, PedsQL is certainly a good tool in clinical practice; however, it does not provide a complete assessment of the many specific psycho-social variables that contribute to the perception of QoL, represented for example by familial socio-economic status, family support, parental stress, anxiety, support networks, and family functioning. This should be elucidated in further studies.

## 5. Conclusions

This is the first study aimed at assessing the impact of life-long treatment with KD on HRQL in a cohort of patients with GLUT1DS.

From the analysis of our sample, we outlined in patients with GLUT1DS a quality of life perception that is comparable to that of other patients with chronic conditions.

In our sample, the presence of movement disorder seems to be a crucial element in quality of life perception.

We also consider it essential to implement psychological support for the patient and for the families in order to improve the quality of life.

Further perspectives studies are needed to better investigate quality of life perception trajectories over time.

## Figures and Tables

**Table 1:** Main characteristics of the study population.

ID	Age	Seizures	MD	ID	KD Ratio	Concomitant AED	PedsQoL Score	Parents	Patient
**pt 1**	4	absent	absent	absent	3.0:1	no	Total functioning	94	NA
							health functioning	*97*	*NA*
							emotional functioning	*97*	*NA*
							social functioning	*100*	*NA*
							school functioning	*92*	*NA*
**pt 2**	11	absent	absent	mild	3.0:1	no	Total functioning	73	74
							health functioning	*69*	*81*
							emotional functioning	*80*	*90*
							social functioning	*65*	*65*
							school functioning	*80*	*55*
**pt 3**	15	absent	daily	moderate	3.0:1	no	Total functioning	60	61
							health functioning	56	63
							emotional functioning	100	95
							social functioning	45	50
							school functioning	40	35
**pt 4**	22	absent	less than monthly	mild	3.0:1	no	Total functioning	47	51
							health functioning	72	75
							emotional functioning	30	35
							social functioning	30	50
							school functioning	40	40
**pt 5**	16	absent	absent	mild	3.0:1	LTG	Total functioning	52	50
							health functioning	44	41
							emotional functioning	50	50
							social functioning	40	40
							school functioning	80	75
**pt 6**	5	absent	absent	mild	3.0:1	no	Total functioning	61	70
							health functioning	56	69
							emotional functioning	65	70
							social functioning	80	80
							school functioning	45	60
**pt 7**	7	absent	absent	absent	2.0:1	no	Total functioning	74	61
							health functioning	75	56
							emotional functioning	85	70
							social functioning	70	70
							school functioning	65	50
**pt 8**	13	absent	less than monthly	absent	2.0:1	no	Total functioning	92	97
							health functioning	94	97
							emotional functioning	100	100
							social functioning	100	100
							school functioning	75	90
**pt 9**	3	absent	weekly	absent	3.0:1	no	Total functioning	58	NA
							health functioning	63	NA
							emotional functioning	50	NA
							social functioning	65	NA
							school functioning	50	NA
**pt 10**	3	absent	less than monthly	mild	2.0:1	no	Total functioning	43	NA
							health functioning	10	NA
							emotional functioning	80	NA
							social functioning	70	NA
							school functioning	15	NA
**pt 11**	19	absent	monthly	mild	3.0:1	VPA	Total functioning	66	68
							health functioning	72	69
							emotional functioning	65	80
							social functioning	70	70
							school functioning	55	55
**pt 12**	13	absent	absent	absent	2.0:1	no	Total functioning	74	76
							health functioning	75	78
							emotional functioning	90	90
							social functioning	65	70
							school functioning	65	65
**pt 13**	13	absent	absent	moderate	3.0:1	no	Total functioning	52	52
							health functioning	66	66
							emotional functioning	65	70
							social functioning	10	5
							school functioning	60	60
**pt 14**	12	absent	absent	mild	3.0:1	no	Total functioning	87	64
							health functioning	100	72
							emotional functioning	90	55
							social functioning	80	75
							school functioning	70	50
**pt 15**	7	absent	daily	mild	4.0:1	no	Total functioning	48	NA
							health functioning	41	NA
							emotional functioning	80	NA
							social functioning	50	NA
							school functioning	25	NA
**pt 16**	4	absent	less than monthly	NA	3.0:1	no	Total functioning	64	NA
							health functioning	60	NA
							emotional functioning	65	NA
							social functioning	60	NA
							school functioning	50	NA
**pt 17**	6	less than monthly	daily	absent	4.0:1	no	Total functioning	53	57
							health functioning	41	44
							emotional functioning	70	80
							social functioning	65	60
							school functioning	45	50

**Table 2:** *p*-value results of the correlation analysis.

	Child Self Report				Parent Proxy Report			
	*Age*	*KD* *Ratio*	*MD* *Frequency*	*ID*	*Age*	*KD* *Ratio*	*MD* *Frequency*	*ID*
**Health/Physical Functioning**	0.81	0.31	0.68	0.61	0.36	0.09	**0.046**	0.19
1. Hard to walk more than a block	0.84	0.2	0.76	0.79	0.35	0.47	0.15	0.68
2. Hard to run	0.79	0.1	0.74	0.53	0.32	0.61	0.25	0.84
3. Hard to sports or exercise	0.87	0.92	0.8	0.23	0.9	0.38	0.94	0.54
4. Hard to lift something heavy	0.43	0.76	0.93	0.72	0.3	0.9	0.57	0.41
5. Hard to take a bath or shower	0.63	0.11	0.56	0.27	0.35	0.12	0.88	0.13
6. Hard to chores around house	0.98	0.73	0.33	**0.01**	0.62	0.55	0.44	**<0.009**
7. Hurt or Ache	0.83	0.39	0.66	0.77	0.22	**0.009**	0.95	0.70
8. Low energy	0.19	0.63	0.69	0.16	0.94	0.2	0.41	0.44
**Emotional Functioning**	0.67	0.51	0.2	0.5	0.76	0.11	0.86	0.49
1. Feel afraid or scared	0.92	0.57	0.60	0.84	0.99	0.51	0.55	0.29
2. Feel sad or blue	0.12	0.74	0.91	0.96	0.28	0.49	0.22	0.67
3. Feel angry	0.92	0.69	0.38	0.32	0.84	0.43	0.52	0.71
4. Trouble sleeping	0.36	0.06	**0.016**	0.65	0.14	0.06	**0.006**	0.42
5. Worry about what will happen	0.64	0.63	0.14	0.25	**0.05**	0.78	0.8	0.6
**Social Functioning**	0.14	0.57	0.72	0.08	0.08	0.32	0.38	**0.045**
1. Trouble getting along with peers	0.23	0.58	0.84	0.23	**0.02**	0.47	0.75	0.08
2. Other kids not wanting to be friends	0.14	0.3	0.44	0.23	0.11	0.78	0.5	**0.02**
3. Teased	0.35	0.87	0.84	**0.007**	0.52	0.58	0.8	**0.01**
4. Doing things other peers do	0.57	0.12	0.87	0.1	0.19	0.07	0.55	0.11
5. Hard to keep up when play with others	0.3	0.09	0.79	0.28	0.62	0.32	0.97	0.17
**School Functioning**	0.92	0.13	0.15	0.41	0.62	0.16	**0.002**	0.2
1. Hard to concentrate	0.94	**0.026**	0.42	0.42	0.71	**0.031**	0.29	0.056
2. Forget things	0.69	0.84	0.87	0.87	0.82	0.35	0.53	**0.05**
3. Trouble keeping up school/work	0.47	0.77	0.68	**0.006**	0.58	**0.01**	0.06	0.14
4. Miss school- Not well	0.72	0.47	0.8	0.94	0.60	0.41	0.27	0.96
5. Miss school- Doctor appointement	0.88	0.41	0.64	0.95	0.90	0.6	**<0.01**	0.63
